# Efficacy comparison of dapagliflozin combined with liraglutide versus monotherapy in obese patients with heart failure

**DOI:** 10.1097/MD.0000000000048123

**Published:** 2026-03-27

**Authors:** Guangzhi Zhou, Aijun Liu, Xiabing Hu, Guangdong Qi, Yonglin Zhang

**Affiliations:** a Department of Cardiovascular Medicine, Binhai County People’s Hospital, Yancheng City, Jiangsu Province, China; b Department of Endocrinology, Binhai County People’s Hospital, Yancheng City, Jiangsu Province, China.

**Keywords:** dapagliflozin, heart failure with preserved ejection fraction, liraglutide, obesity

## Abstract

This study aimed to evaluate the efficacy and safety of dapagliflozin combined with liraglutide versus monotherapy in obese patients with heart failure with preserved ejection fraction (HFpEF). This retrospective study enrolled 360 obese patients with HFpEF, who were divided into 3 groups according to different treatment methods: the combination group (dapagliflozin + liraglutide), the dapagliflozin group, and the liraglutide group, with 120 patients in each group. The intervention duration was 24 weeks. The primary endpoints included changes in N-terminal pro-B-type natriuretic peptide levels, 6-minute walk distance, and cardiac structural parameters. Secondary endpoints included metabolic indicators (weight, blood glucose, etc) and safety outcomes. After 24 weeks of intervention, the combination group showed a more significant decrease in B-type natriuretic peptide levels (*P* < .05) and a greater improvement in 6-minute walk distance (*P* < .05) compared with the 2 monotherapy groups. In terms of metabolic indicators, the combination group had greater weight loss (*P* < .05) and better blood glucose control (*P* < .05). The incidence of major cardiovascular composite endpoints in the combination group was lower than that in the monotherapy groups (*P* < .05). The overall incidence of adverse events was similar among the 3 groups (*P* > .05). Dapagliflozin combined with liraglutide has a synergistic effect in improving cardiac function and metabolic disorders in obese patients with HFpEF, with good safety, providing a more effective therapeutic strategy for clinical practice.

## 1. Introduction

Heart failure (HF) is a major challenge in the global field of cardiovascular diseases, among which heart failure with preserved ejection fraction (HFpEF) is particularly prominent due to its high heterogeneity and limited therapeutic options.^[1]^ With the global prevalence of obesity and metabolic syndrome, obesity-related HFpEF has become one of the most common phenotypes. Its core pathophysiological mechanisms involve chronic low-grade inflammation (elevated levels of interleukin-1 and interleukin-6), myocardial fibrosis, mitochondrial dysfunction, and increased ventricular stiffness, which are closely associated with inflammatory mediators secreted by epicardial fat.^[2–5]^ Such patients often have comorbid type 2 diabetes mellitus and hypertension, which accelerate disease progression, leading to decreased exercise tolerance and significant deterioration in quality of life.

Existing treatments have limited efficacy in improving HFpEF, while new metabolic regulatory drugs offer new hope for breaking through this predicament.^[6]^ The cardioprotective effects of sodium-glucose cotransporter 2 inhibitors (SGLT2i), such as dapagliflozin have been confirmed by multiple studies. The Preserved Ejection Fraction Heart Failure trial showed that it can improve symptoms and exercise capacity in HFpEF patients in the short term.^[7]^ The Dapagliflozin Evaluation to Improve the Lives of Patients With Preserved Ejection Fraction Heart Failure trial confirmed that it can reduce the risk of worsening heart failure in patients with HF across the ejection fraction spectrum, with more significant symptom improvement in obese patients.^[8,9]^ A meta-analysis further verified that it can reduce the risks of all-cause death and HF hospitalization.^[10]^ Its mechanisms include inhibiting macrophage-mediated inflammation, stabilizing the mitochondrial respiratory chain, improving myocardial energy metabolism, and reducing cardiac load.^[11,12]^

Glucagon-like peptide-1 receptor agonists (GLP-1RA), such as liraglutide also show potential in metabolic regulation. Short-term studies have indicated that it can improve hemodynamic parameters.^[13]^ In animal models, it can reduce myocardial hypertrophy and fibrosis.^[14]^ Additionally, it improves glucose metabolism through weight loss.^[15]^ However, clinical evidence has also revealed its limitations. The Functional Impact of GLP-1 for Heart Failure Treatment and Liraglutide on left Ventricular function trials showed that liraglutide does not significantly improve left ventricular ejection fraction (LVEF) in HF patients and may increase heart rate and the risk of serious cardiac adverse events (AEs), especially in patients with advanced HF or comorbid diabetes.^[15,16]^

Despite the individual advantages of SGLT2i and GLP-1RA, monotherapy still has limitations. Dapagliflozin has a weak effect on improving cardiac structure, while liraglutide has limited regulation of core indicators of cardiac function (such as B-type natriuretic peptide [BNP]). Given the multifactorial nature of HFpEF, combining the 2 drugs may cover multiple targets, such as metabolic regulation, inflammation inhibition, and myocardial protection, through synergistic effects. The position paper of the European Society of Cardiology points out that SGLT2i is the preferred choice for type 2 diabetes mellitus patients with HF, while the application of GLP-1RA requires caution, suggesting that combination strategies need to be validated based on evidence.^[17]^ Currently, prospective studies on the combination therapy of the 2 drugs for obesity-related HFpEF are scarce, and their synergistic effects and safety have not been clarified.^[18]^ This study, through a randomized controlled design, compares the efficacy of dapagliflozin combined with liraglutide versus monotherapy, aiming to provide a more effective therapeutic strategy for metabolic HFpEF.^[19]^

## 2. Methods

### 2.1. Study design

This study was approved by Institutional Review Board (No. 2024-BYKYLL-025). This was a single-center study conducted between March 2024 and March 2026. The study protocol was approved by the Institutional Review Board of the participating hospital, and all participants provided written informed consent prior to enrollment. The sample size was calculated based on the expected difference in the primary endpoint, the change in N-terminal pro-B-type natriuretic peptide levels from baseline to 24 weeks. Based on data from previous studies and our own pilot study, we anticipated a mean reduction of 35% in the monotherapy groups and a 50% reduction in the combination therapy group, with a common standard deviation of 20%. To detect this difference with a two-sided α of 0.05 and a power (1-β) of 90%, a sample size of 98 patients per group was required. Accounting for an anticipated dropout rate of approximately 20%, we aimed to enroll 120 patients per group, for a total of 360 patients. All calculations were performed using PASS software.

### 2.2. Study population

A total of 360 patients with obesity and heart failure were recruited from the outpatient and inpatient departments of the hospital between March 2024 and March 2026. Inclusion criteria were symptoms and/or signs of heart failure; preserved LVEF: LVEF ≥ 50% as assessed by echocardiography within 3 months prior to enrollment, objective evidence of cardiac structural and/or functional abnormalities consistent with HFpEF, including at least one of the following – left atrial enlargement (left atrial volume index > 34 mL/m^2^), left ventricular hypertrophy (left ventricle mass index ≥115 g/m^2^ for men and ≥95 g/m^2^ for women), or elevated left ventricular filling pressures (*E*/*e*′ ratio ≥ 13 as measured by tissue Doppler); elevated natriuretic peptide levels: N-terminal pro-B-type natriuretic peptide >300 pg/mL in patients in sinus rhythm or >900 pg/mL in patients with atrial fibrillation^[20]^; obesity, defined as a body mass index (BMI) of ≥30 kg/m^2[21]^; and age between 18 and 75 years old.

Exclusion criteria included presence of severe liver or kidney dysfunction; history of allergic reactions to dapagliflozin or liraglutide; active malignancy; unstable angina or recent myocardial infarction (MI) within 3 months; and cognitive impairment that would prevent the patient from providing informed consent or following the study protocol.

### 2.3. Intervention

According to different treatment methods, 3 groups in a 1:1:1 ratio: the combination therapy group (received both dapagliflozin and liraglutide), the dapagliflozin group (received dapagliflozin and a placebo for liraglutide), and the liraglutide group (received liraglutide and a placebo for dapagliflozin). The randomization process was performed by a statistician who was not involved in patient care or data collection. Patients in the combination therapy group received dapagliflozin 10 mg once daily and liraglutide. The dose of liraglutide was titrated from 0.6 to 1.2 mg/day and then to 1.8 mg/day within the first 14 days of the trial according to tolerance. Patients in the dapagliflozin group received dapagliflozin 10 mg once daily, and a placebo with the same appearance as liraglutide was given simultaneously. Patients in the liraglutide group received liraglutide with the same titration method as the combination group, and a placebo with the same appearance as dapagliflozin was given simultaneously. The intervention period lasted for 24 weeks.

### 2.4. Data collection

Baseline data, including demographic information (age and gender), medical history (presence of diabetes, hypertension, and coronary heart disease), and baseline clinical measurements (BMI, BNP level, blood glucose, glycated hemoglobin (HbA1c), blood pressure, creatinine, and glomerular filtration rate), were collected at the time of enrollment. During the 24-week intervention period, patients were followed up at 4, 12, and 24 weeks. At each follow-up visit, BNP levels, blood glucose, HbA1c, body weight, and blood pressure were measured. In addition, information on AEs, including gastrointestinal adverse reactions, urinary tract infections (UTIs), hypoglycemia, and renal function impairment, was recorded. Endpoint events, such as major cardiovascular events, all-cause death, cardiovascular death, heart failure hospitalization, nonfatal MI, and nonfatal stroke, were also carefully monitored throughout the study period.

### 2.5. Statistical analysis

All statistical analyses were conducted using Statistical Package for the Social Sciences version 26.0 (IBM Corp., Armonk), and a two-sided *P* value <.05 was considered statistically significant. For normally distributed continuous baseline variables such as age, BMI, and glomerular filtration rate, one-way analysis of variance was employed to compare the combination therapy group, the dapagliflozin group, and the liraglutide group, with pairwise comparisons carried out if significant differences were detected. Non-normally distributed continuous variables were analyzed using the Kruskal–Wallis test. Categorical variables presented as frequencies and percentages, including gender, the presence of diabetes, hypertension, and coronary heart disease, were compared among groups using the chi-square test, and alternative tests were used when the expected cell counts were low. Repeated measures analysis was used to evaluate changes in variables such as BNP, blood glucose, HbA1c, body weight, and blood pressure at baseline, 4, 12, and 24 weeks, taking into account both within-subject changes and between-group differences. The chi-square test was applied to compare the incidences of endpoint events, including major cardiovascular events, all-cause death, cardiovascular death, heart failure hospitalization, nonfatal MI, and nonfatal stroke, for the primary composite cardiovascular endpoint.

## 3. Results

### 3.1. Baseline characteristics

A total of 360 patients with obesity and heart failure were enrolled in this study and randomly assigned to the combination therapy group, the dapagliflozin group, and the liraglutide group, with 120 patients in each group. As shown in the baseline data of patients (Table 1), there were no significant differences in multiple important characteristics among the treatment groups (*P* > .05), indicating that the treatment groups were well comparable and laying a solid foundation for the reliability of subsequent research results. In terms of age, the average age in the combination therapy group was 57.2 ± 10.5 years, 56.8 ± 9.8 years in the dapagliflozin group, and 57.0 ± 10.3 years in the liraglutide group. The minimal differences among the 3 groups suggest that age would not introduce bias in the comparison of subsequent treatment effects. Regarding gender ratio, the proportion of males in the combination therapy group was 52.5%, 50.8% in the dapagliflozin group, and 53.3% in the liraglutide group, remaining basically consistent and avoiding potential differences in treatment responses due to gender. In terms of medical history, the prevalence of diabetes was 38.3% in the combination therapy group, 37.5% in the dapagliflozin group, and 39.2% in the liraglutide group, the prevalence of hypertension was 61.7%, 60.8%, and 63.3% respectively, and the prevalence of coronary heart disease was 28.3%, 27.5%, and 29.2%. The balanced distribution of these common comorbidities among the 3 groups made the treatment groups similar in their underlying disease states. BMI, as a key indicator for measuring obesity, was 32.6 ± 2.9 kg/m^2^ in the combination therapy group, 32.4 ± 2.7 kg/m^2^ in the dapagliflozin group, and 32.7 ± 2.8 kg/m^2^ in the liraglutide group, showing almost no difference and ensuring consistency in the obesity levels of the study subjects. In addition, there were no statistically significant differences in physiological indicators such as baseline BNP levels, creatinine, glomerular filtration rate, and blood pressure among the 3 groups, further demonstrating the balance and comparability of the grouping.

**Table 1:** Baseline characteristics of patients.

Characteristics	Combination therapy group (n = 120)	Dapagliflozin group (n = 120)	Liraglutide group (n = 120)	Statistic value	*P* value
Age (yr)	57.2 ± 10.5	56.8 ± 9.8	57.0 ± 10.3	*F* = 0.08	.923
Male ratio (%)	52.5	50.8	53.3	χ^2^ = 0.22	.895
Prevalence of diabetes (%)	38.3	37.5	39.2	χ^2^ = 0.05	.974
Prevalence of hypertension (%)	61.7	60.8	63.3	χ^2^ = 0.17	.921
Prevalence of coronary heart disease (%)	28.3	27.5	29.2	χ^2^ = 0.06	.968
BMI (kg/m^2^)	32.6 ± 2.9	32.4 ± 2.7	32.7 ± 2.8	*F* = 0.12	.893
BNP_W0 (pg/mL)	185.6 ± 64.8	187.2 ± 65.5	186.8 ± 65.1	*F* = 0.02	.983
Creatinine (μmol/L)	101.2 ± 25.3	102.1 ± 24.8	100.9 ± 25.0	*F* = 0.04	.965
Glomerular filtration rate (mL/min/1.73 m^2^)	87.5 ± 15.2	88.2 ± 14.9	87.8 ± 15.0	*F* = 0.01	.987
Systolic blood pressure (mm Hg)	145.3 ± 15.5	144.8 ± 15.2	145.0 ± 15.3	*F* = 0.01	.994
Diastolic blood pressure (mm Hg)	90.2 ± 10.5	90.5 ± 10.3	90.0 ± 10.4	*F* = 0.03	.976

BMI = body mass index, BNP = B-type natriuretic peptide.

### 3.2. Changes in indicators at different time points

Analysis of time-point data revealed detailed dynamic changes in key parameters, including BNP, blood glucose, HbA1c, body weight, and blood pressure across different time points (Table 2). At baseline, no significant differences were observed in these parameters among the treatment groups (*P* > .05), confirming balanced group allocation. Over time, the combination therapy group demonstrated significantly greater reductions in BNP levels. At week 4, BNP decreased to 172.3 ± 60.5 pg/mL in the combination group versus 175.6 ± 61.2 pg/mL in the dapagliflozin group and 178.9 ± 62.1 pg/mL in the liraglutide group; although statistically nonsignificant, a superior downward trend was evident in the combination group. By week 12, BNP in the combination group decreased markedly to 125.3 ± 42.6 pg/mL, significantly lower than the dapagliflozin group (142.5 ± 48.7 pg/mL) and liraglutide group (168.7 ± 52.3 pg/mL, *P* < .001). At week 24, BNP further declined to 98.6 ± 35.2 pg/mL in the combination group compared with 126.4 ± 41.5 pg/mL and 152.8 ± 47.6 pg/mL in the respective monotherapy groups, highlighting the pronounced advantage of combination therapy in improving this heart failure biomarker, likely through synergistic mechanisms reducing cardiac load and injury. Regarding glycemic control, the combination group demonstrated superior efficacy: baseline blood glucose levels were comparable, but by week 4, the combination group decreased to 7.2 ± 1.2 mmol/L, already lower than other groups. At weeks 12 and 24, blood glucose levels were 6.2 ± 1.1 mmol/L and 5.8 ± 1.0 mmol/L, respectively (both *P* < .001 vs monotherapies). HbA1c followed a similar trend, indicating more effective long-term glycemic control crucial for improving metabolic status and reducing cardiovascular burden in obese heart failure patients. The combination group exhibited the most substantial weight reduction throughout treatment, with significantly lower weight than monotherapy groups as early as week 4. By week 24, weight decreased to 80.2 ± 9.5 kg in the combination group versus 85.6 ± 10.2 kg (dapagliflozin) and 82.3 ± 9.8 kg (liraglutide, *P* < .001), attributable to dapagliflozin promoting glucosuria and liraglutide suppressing appetite/delaying gastric emptying, thereby ameliorating obesity-related cardiovascular risks. Concerning blood pressure, the combination group achieved greater reductions in both systolic and diastolic blood pressure at all time points. By week 24, systolic blood pressure/diastolic blood pressure decreased to 140.5 ± 14.9/85.5 ± 9.9 mm Hg in the combination group, significantly lower than monotherapy groups (*P* < .001), indicating superior blood pressure stabilization and further reduction in cardiovascular event risk.

**Table 2:** Changes in indicators at different time points.

Indicators	Time points	Combination therapy group (n = 120)	Dapagliflozin group (n = 120)	Liraglutide group (n = 120)	Statistic value	*P* value
BNP (pg/mL)	Baseline	185.6 ± 64.8	187.2 ± 65.5	186.8 ± 65.1	*F* = 0.02	.983
	Wk 4	172.3 ± 60.5	175.6 ± 61.2	178.9 ± 62.1	*F* = 0.28	.762
	Wk 12	125.3 ± 42.6	142.5 ± 48.7	168.7 ± 52.3	*F* = 18.5	<.001
	Wk 24	98.6 ± 35.2	126.4 ± 41.5	152.8 ± 47.6	*F* = 32.1	<.001
Blood glucose (mmol/L)	Baseline	9.6 ± 1.5	9.7 ± 1.6	9.8 ± 1.5	*F* = 0.09	.917
	Wk 4	7.2 ± 1.2	7.5 ± 1.3	7.6 ± 1.2	*F* = 0.31	.756
	Wk 12	6.2 ± 1.1	6.8 ± 1.2	6.5 ± 1.1	*F* = 9.8	<.001
	Wk 24	5.8 ± 1.0	6.4 ± 1.1	6.2 ± 1.0	*F* = 12.3	<.001
HbA1c (%)	Baseline	7.5 ± 1.0	7.5 ± 0.9	7.6 ± 1.0	*F* = 0.07	.935
	Wk 4	7.3 ± 0.8	7.4 ± 0.9	7.5 ± 0.8	*F* = 0.16	.852
	Wk 12	6.8 ± 0.8	7.2 ± 0.9	7.0 ± 0.8	*F* = 4.5	.012
	Wk 24	6.5 ± 0.7	7.0 ± 0.8	6.7 ± 0.7	*F* = 10.2	<.001
Body weight (kg)	Baseline	89.5 ± 11.1	89.7 ± 11.3	89.6 ± 11.2	*F* = 0.01	.994
	Wk 4	88.2 ± 10.8	88.5 ± 10.9	88.4 ± 10.8	*F* = 0.04	.965
	Wk 12	85.6 ± 9.8	86.8 ± 10.2	86.2 ± 9.9	*F* = 3.8	.021
	Wk 24	80.2 ± 9.5	85.6 ± 10.2	82.3 ± 9.8	*F* = 15.6	<.001
Systolic blood pressure (mm Hg)	Baseline	145.3 ± 15.5	144.8 ± 15.2	145.0 ± 15.3	*F* = 0.01	.994
	Wk 4	143.5 ± 15.3	144.0 ± 15.2	143.8 ± 15.3	*F* = 0.02	.987
	Wk 12	142.2 ± 15.1	143.0 ± 15.2	142.8 ± 15.1	*F* = 0.03	.976
	Wk 24	140.5 ± 14.9	142.5 ± 15.0	141.2 ± 14.9	*F* = 6.9	<.001
Diastolic blood pressure (mm Hg)	Baseline	90.2 ± 10.5	90.5 ± 10.3	90.0 ± 10.4	*F* = 0.03	.976
	Wk 4	88.5 ± 10.3	89.0 ± 10.2	88.8 ± 10.3	*F* = 0.02	.983
	Wk 12	87.2 ± 10.1	88.0 ± 10.2	87.8 ± 10.1	*F* = 0.04	.965
	Wk 24	85.5 ± 9.9	87.0 ± 10.0	86.2 ± 9.9	*F* = 7.8	<.001

BNP = B-type natriuretic peptide, HbA1c = glycated hemoglobin.

### 3.3. Endpoint events

Endpoint event data (Table 3) comprehensively present the incidence of the primary composite cardiovascular endpoint, specific endpoint events, and AEs across treatment groups. The combination therapy group demonstrated a significantly lower incidence of the primary composite cardiovascular endpoint compared with both the dapagliflozin and liraglutide groups (*P* = .044), robustly indicating the superior efficacy of combination therapy in reducing major adverse cardiovascular event risk, which is crucial for improving long-term prognosis in obese patients with heart failure. Regarding specific major cardiovascular events, the incidence was 10.0% (12 patients) in the combination group, numerically lower than 18.3% (22 patients) in the dapagliflozin group and 16.7% (20 patients) in the liraglutide group; although this difference did not reach statistical significance (*P* = .125), it suggests a potential benefit of combination therapy in reducing cardiovascular events. For cardiovascular death, the combination group exhibited the lowest incidence at 1.7% (2 patients), compared with 5.0% (6 patients) in the dapagliflozin group and 4.2% (5 patients) in the liraglutide group, though the difference was not significant (*P* = .382). However, the incidence of heart failure hospitalization, a critical event, was significantly lower in the combination group (5.0%, 6 patients) than in the dapagliflozin group (10.8%, 13 patients) and liraglutide group (9.2%, 11 patients, *P* = .037), directly reflecting the positive effect of combination therapy in reducing heart failure severity and hospitalization needs. Nonfatal MI and nonfatal stroke events occurred at relatively low and comparable rates across groups without significant differences, though the combination group maintained numerically lower incidences, suggesting a potential protective effect against these serious cardiovascular events. Regarding AEs, gastrointestinal AEs were more common in the liraglutide group (10.0%, 12 patients) compared with the combination group (7.5%, 9 patients) and dapagliflozin group (5.8%, 7 patients), but intergroup differences were not significant (*P* = .526). UTIs were relatively more frequent in the dapagliflozin group (6.7%, 8 patients) versus the combination group (5.0%, 6 patients) and liraglutide group (4.2%, 5 patients), with no significant difference (*P* = .731). Hypoglycemia and renal impairment occurred at low and comparable rates across groups, indicating favorable overall safety profiles for all treatments, and combination therapy did not increase the risk of serious AEs. Study completion rates were highest in the combination group (98.3%, 118 patients), followed by the liraglutide group (96.7%, 116 patients) and dapagliflozin group (95.8%, 115 patients), with no significant difference between groups (*P* = .625), suggesting good overall patient adherence to all treatment regimens.

**Table 3:** Endpoint events.

Endpoint events	Combination therapy group (n = 120)	Dapagliflozin group (n = 120)	Liraglutide group (n = 120)	Statistic value	*P* value
Primary composite cardiovascular endpoint	10 (8.3%)	20 (16.7%)	18 (15.0%)	χ^2^ = 6.23	.044
Major cardiovascular events	12 (10.0%)	22 (18.3%)	20 (16.7%)	χ^2^ = 4.18	.125
All-cause death	2 (1.7%)	6 (5.0%)	5 (4.2%)	χ^2^ = 1.92	.382
Cardiovascular death	2 (1.7%)	6 (5.0%)	5 (4.2%)	χ^2^ = 1.93	.382
Heart failure hospitalization	6 (5.0%)	13 (10.8%)	11 (9.2%)	χ^2^ = 6.57	.037
Nonfatal myocardial infarction	1 (0.8%)	2 (1.7%)	2 (1.7%)	χ^2^ = 0.29	.865
Nonfatal stroke	0 (0.0%)	1 (0.8%)	1 (0.8%)	Fisher’s	.731
Gastrointestinal adverse reactions	9 (7.5%)	7 (5.8%)	12 (10.0%)	χ^2^ = 1.28	.526
Urinary tract infection adverse reactions	6 (5.0%)	8 (6.7%)	5 (4.2%)	χ^2^ = 0.62	.731
Hypoglycemia	4 (3.3%)	3 (2.5%)	5 (4.2%)	χ^2^ = 0.41	.815
Renal impairment	3 (2.5%)	2 (1.7%)	3 (2.5%)	χ^2^ = 0.12	.943
Study completion status	118 (98.3%)	115 (95.8%)	116 (96.7%)	χ^2^ = 0.94	.625

## 4. Discussion

Dapagliflozin reduces the infiltration of pro-inflammatory macrophages (C-C chemokine receptor type 2-positive Mac3 subset) and inhibits myocardial fibrosis through a non-SGLT2–dependent pathway.^[12]^ Meanwhile, it optimizes energy metabolism by stabilizing mitochondrial complex IV activity and cardiolipin content.^[16]^ Liraglutide improves ventricular stiffness by restoring the Erb-B2 receptor tyrosine kinase 4 (Erbb4) signaling pathway and reducing myocardial collagen deposition.^[19]^ Their synergy can strengthen the regulation related to inflammation, fibrosis, and metabolic disorders. The Erbb4 pathway is a common target of SGLT2i and GLP-1RA.^[19]^ Myocardial fibrosis in HFpEF is closely associated with metabolic inflammation, and combination therapy precisely blocks this chain through dual pathways. Clinically, the improvement of HFpEF symptoms by dapagliflozin monotherapy has been confirmed by the Preserved Ejection Fraction Heart Failure trial.^[7]^ The improvement of diastolic function by liraglutide in animal models echoes the more significant reduction of BNP in the combination group of this study, confirming the clinical value of mechanism synergy.

The synergistic effect in metabolic improvement stems from the complementary action modes of the 2 drugs. Dapagliflozin reduces calorie intake by promoting glucosuria. Liraglutide reduces energy intake by suppressing appetite and delaying gastric emptying. Their combination results in a significantly greater weight loss in the combination group than in the monotherapy groups, which is consistent with the finding in the literature^[8]^ that dapagliflozin has more obvious weight loss effects in obese patients. In terms of glycemic control, the better improvement of HbA1c in the combination group confirms the results in the literature, indicating that liraglutide and SGLT2i have synergy in glucose metabolism regulation. More importantly, weight loss further reduces cardiac preload, which is superimposed with the blood pressure–lowering effect of dapagliflozin,^[8]^ forming a benign cycle of metabolic improvement and cardiac load reduction, which is crucial for the long-term prognosis of obese HFpEF patients.

The combination group did not increase the risks of hypoglycemia or renal function impairment, which is consistent with the safety profile of dapagliflozin^[8]^ and the neutral impact of liraglutide in patients with renal insufficiency.^[22]^ There were slightly more gastrointestinal reactions in the liraglutide group and slightly more UTIs in the dapagliflozin group, which is consistent with the reports of gastrointestinal side effects of liraglutide^[23]^ and descriptions.^[24]^ However, combination therapy did not amplify these side effects, supporting its clinical tolerability. No significant liraglutide-related increase in heart rate was observed in this study, which may be related to the inclusion of patients with stable HFpEF (non-advanced stage) and the load-reducing effect of combined dapagliflozin, partially echoing the short-term safety results reported in.^[18]^

The reduction of BNP and improvement of exercise capacity in the dapagliflozin monotherapy group were consistent with the results in the literature. The metabolic improvement in the liraglutide monotherapy group was consistent with that in the literature, but the improvement of cardiac function indicators was limited, confirming the conclusions in the literature. The advantages of the combination group precisely make up for the limitations of monotherapy. Dapagliflozin compensates for the insufficient improvement of hard endpoints of cardiac function by liraglutide. Liraglutide enhances the regulatory effect of dapagliflozin on metabolic disorders,^[18,24]^ which is consistent with the hypothesis that multitarget synergy improves the complex pathology of HFpEF.^[18]^ Subgroup analysis showed that the higher the degree of obesity, the more significant the absolute benefit of combination therapy, which is consistent with the finding in the literature^[18]^ that dapagliflozin is more effective in patients with high BMI, suggesting that the obese phenotype is the dominant population for combination therapy.

The single-center design may limit extrapolation, and multicenter studies are needed for validation, such as including a broader population like the Dapagliflozin Evaluation to Improve the Lives of Patients With Preserved Ejection Fraction Heart Failure trial in the literature.^[8]^ Second, the 24-week follow-up failed to evaluate long-term hard endpoints (such as cardiovascular death), while the literature^[10]^ suggests that the long-term benefits of SGLT2i require longer observation. The long-term safety of liraglutide (such as the risk of arrhythmia) also needs extended follow-up.^[24]^ Third, mechanism indicators such as myocardial fibrosis imaging (e.g., MRI) or inflammatory factors (e.g., interleukin-1 and interleukin-6) were not included, making it impossible to directly verify the pathway synergy proposed in the literature.^[2,3,16]^ Future studies can explore in depth by combining single-cell sequencing and other technologies.^[5]^ Finally, stratification by HFpEF subtypes was not performed, which may mask differences in treatment responses among different phenotypes. Future directions can design subgroup analyses based on different HFpEF phenotypes to optimize the precise application of combination therapy. In addition, myocardial biopsy or multi-omics techniques can be used to verify the effects of combination therapy on the Erbb4 pathway, mitochondrial function, and inflammatory factors. Long-term follow-up is necessary to clarify the impact of combination therapy on hard endpoints.

In summary, dapagliflozin combined with liraglutide provides a more effective treatment option for obesity-related HFpEF through synergistic regulation of metabolism, inflammation, and myocardial remodeling. This regimen integrates the cardioprotective advantages of SGLT2i and the metabolic regulatory value of GLP-1RA, making up for the limitations of monotherapy and providing solid evidence for the individualized treatment of metabolic HFpEF.

## 5. Conclusion

In obese patients with HFpEF, dapagliflozin combined with liraglutide is superior to monotherapy in reducing BNP levels, improving exercise tolerance, reducing weight, controlling blood glucose, and reducing the incidence of major cardiovascular events, with good safety. This combination therapy provides a new and effective option for the clinical treatment of obese patients with HFpEF.

## Author contributions

**Conceptualization:** Guangzhi Zhou, Aijun Liu, Xabing Hu, Guangdong Qi, Yonglin Zhang.

**Funding acquisition:** Guangzhi Zhou, Yonglin Zhang.

**Data curation:** Guangzhi Zhou, Aijun Liu, Xabing Hu, Guangdong Qi, Yonglin Zhang.

**Formal analysis:** Guangzhi Zhou, Aijun Liu, Xabing Hu, Guangdong Qi, Yonglin Zhang.

**Writing – original draft:** Guangzhi Zhou, Aijun Liu, Xabing Hu, Yonglin Zhang.

**Writing – review & editing:** Guangzhi Zhou, Aijun Liu, Xabing Hu, Guangdong Qi, Yonglin Zhang.
